# Supplementary material for: Adenovirus maturation establishes the transcription competent packaging of its genome
Source: EMBO Rep. 2025 Oct 21;26(22):5589–611. doi: 10.1038/s44319-025-00598-z (PMC12635127; doi:10.1038/s44319-025-00598-z)
Supplement: Supplementary file 7 — Expanded View Figures [file 44319_2025_598_MOESM7_ESM.pdf]

Expanded View Figures

Figure EV1. Depiction of DMS-seq workflow and controls.

(**A**) Scheme showing the DMS caused fragmentation of DNA by Putrescine and APE1. (**B**) Agarose gel (1.3%) of viral DNA was treated in the absence or presence of 5% DMS for the indicated time points. (**C**) Viral DNA was fragmented after treatment with 2% DMS for 4 min, and a subsequent cleavage reaction was used for library preparation. (**D**) Aligned fragment size distribution histogram of free DNA, Ad-wt, and Ad-ts1. (**E**) Overview of the pipeline for the processing of DMS-seq data. (**F**) Plot of nucleotide frequencies relative to fragment cleavage positions, as indicated.

© The Author(s)

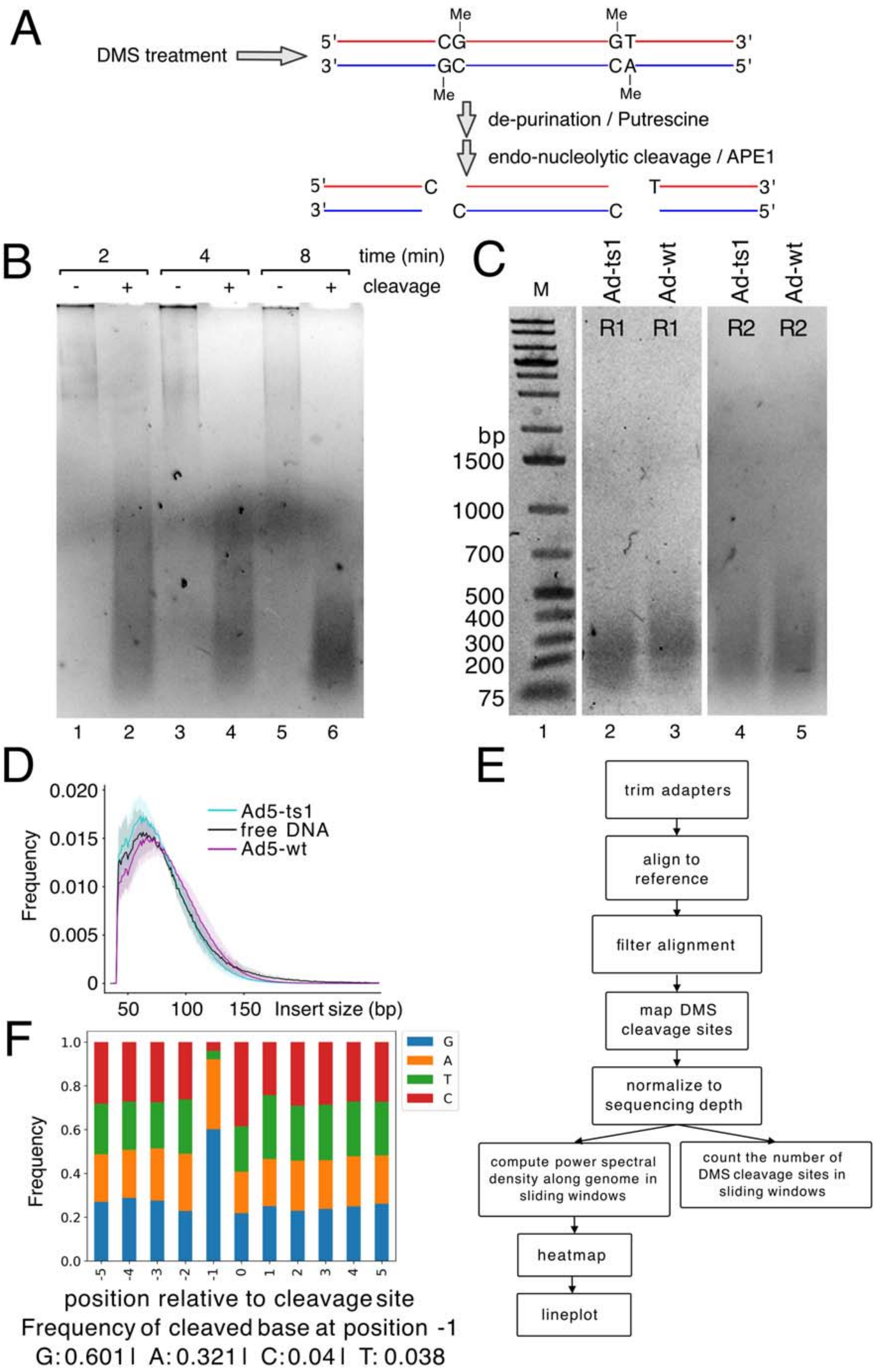
A
DMS treatment
Me
5' CG GT 3'
3' GC CA 5'
de-purination / Putrescine
endo-nucleolytic cleavage / APE1
B
2 4 8 time (min)
- + - + - + cleavage
1 2 3 4 5 6
C
M Ad-ts1 Ad-wt Ad-ts1 Ad-wt
R1 R1 R2 R2
bp
1500
1000
700
500
400
300
200
75
1 2 3 4 5
D
Ad5-ts1
free DNA
Ad5-wt
Frequency
Insert size (bp)
E
trim adapters
align to reference
filter alignment
map DMS cleavage sites
normalize to sequencing depth
compute power spectral density along genome in sliding windows
count the number of DMS cleavage sites in sliding windows
heatmap
lineplot
F
G
A
T
C
Frequency
position relative to cleavage site
Frequency of cleaved base at position -1
G: 0.601 | A: 0.321 | C: 0.04 | T: 0.038

 © The Author(s)

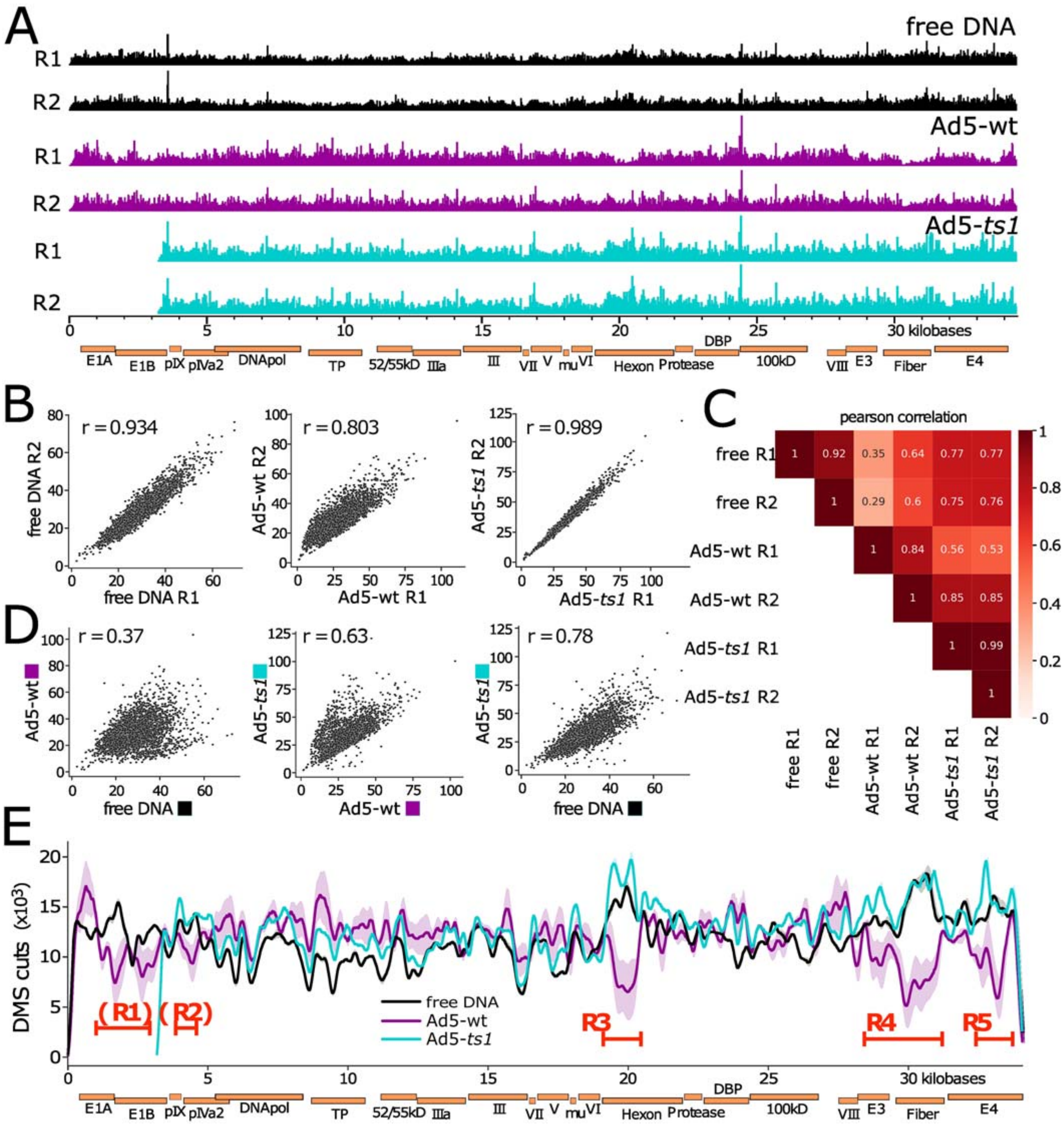

Figure EV2. DMS cleavage site distribution along the genome.

(**A**) Bar chart of cleavage sites along the reference genomes, individually displayed for each replicate. (**B**) Correlation between replicates in 10 bp bins along the genome. (**C**) Correlation matrix for the individual samples generated by deeptools ('multiBamSummary bins -bs 5'). (**D**) Correlation between samples (replicates combined) in 10 bp bins along the genome. (**E**) Count of DMS cut sites in a 400 bp sliding window with 100 bp step size across the reference genomes.

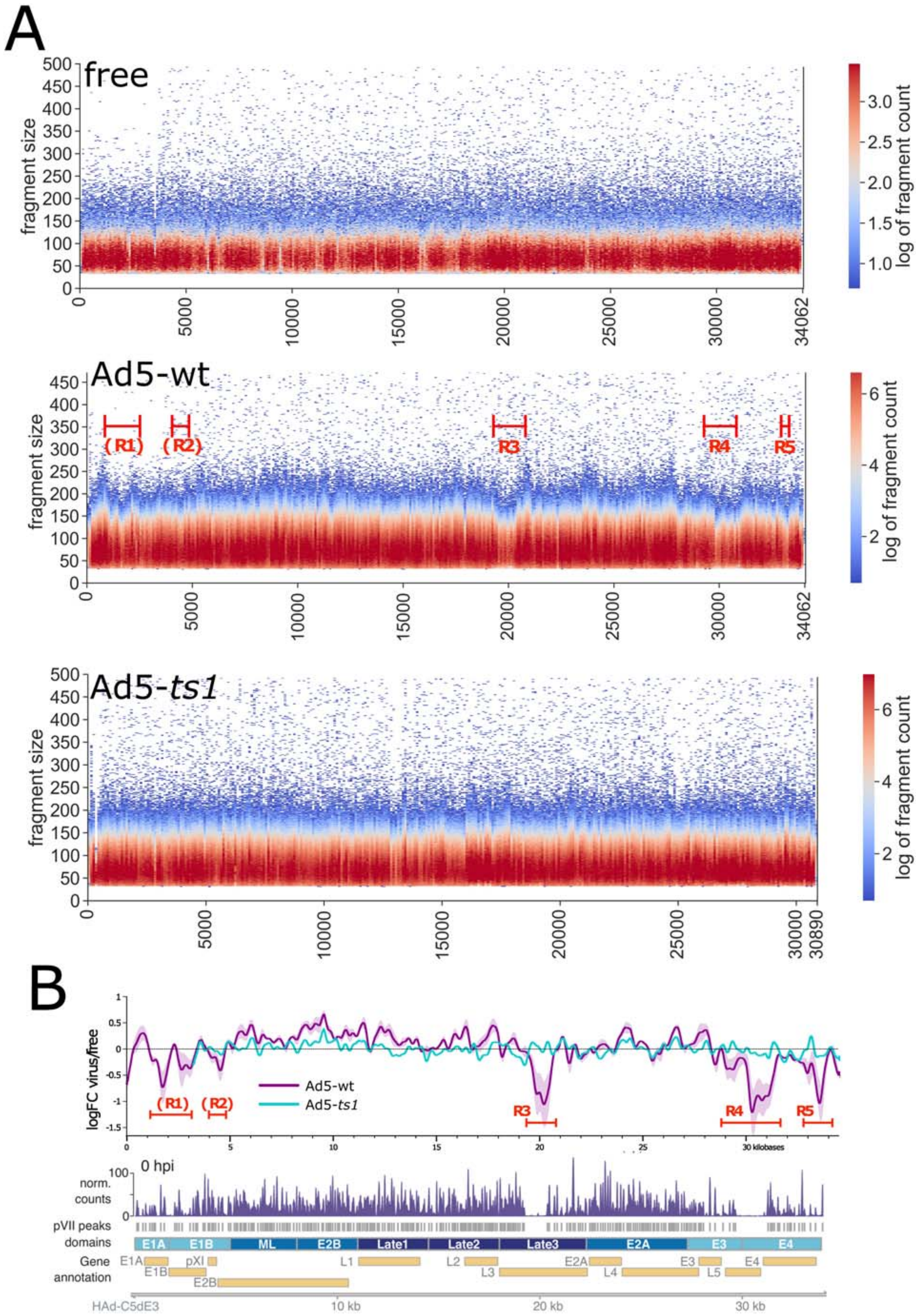
A
free
fragment size
log of fragment count
Ad5-wt
(R1)
(R2)
R3
R4
R5
Ad5-ts1
B
logFC virus/free
Ad5-wt
Ad5-ts1
30 kilobases
0 hpi
norm. counts
pVII peaks
domains
E1A
E1B
ML
E2B
Late1
Late2
Late3
E2A
E3
E4
Gene annotation
pXI
L1
L2
L3
L4
L5
HAd-C5dE3
10 kb
20 kb
30 kb

Figure EV3. DNA size evaluation along the adenoviral genome.

(**A**) V-plots (heatmaps) plotting the fragment size against the fragment midpoint for all samples. (**B**) Comparison of DMS-seq data with MNase-seq coverage along the adenovirus genome. Top panel: log fold change of DMS methylation sites of viral samples against free DNA (Fig. 1C). Bottom panel: MNase-seq coverage (adapted from Schwartz et al, 2023).

© The Author(s)

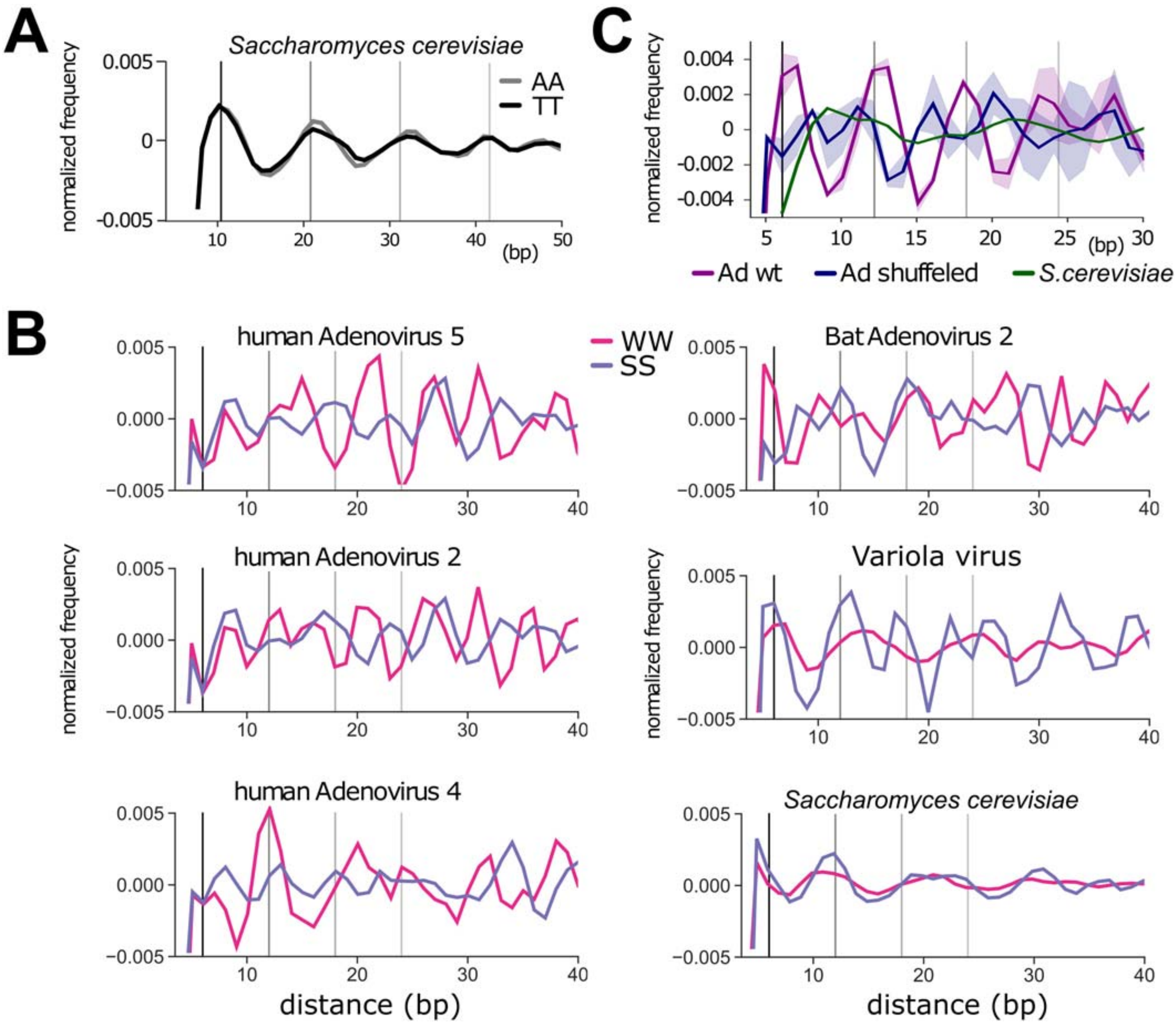

Figure EV4. Dinucleotide distance histograms.

(**A**) Nucleotide repeat patterns of ~11-bp AA and TT on the S. cerevisiae genome. Vertical lines are placed every 10.3 bp. (**B**) SS (G/C) and WW (A/T) dinucleotide sequence distance histograms on genomes from different adenovirus species, including Variola virus and the yeast genome. Vertical lines are placed every 6.1 bp. (**C**) Dinucleotide (~6-bp) periodicity of the T/C (YY) distance histogram on the human adenovirus 5 genome. The same analysis was performed with the shuffled Adenovirus genome and the S. cerevisiae genome. Vertical lines are drawn every 6.1 bp.

 © The Author(s)

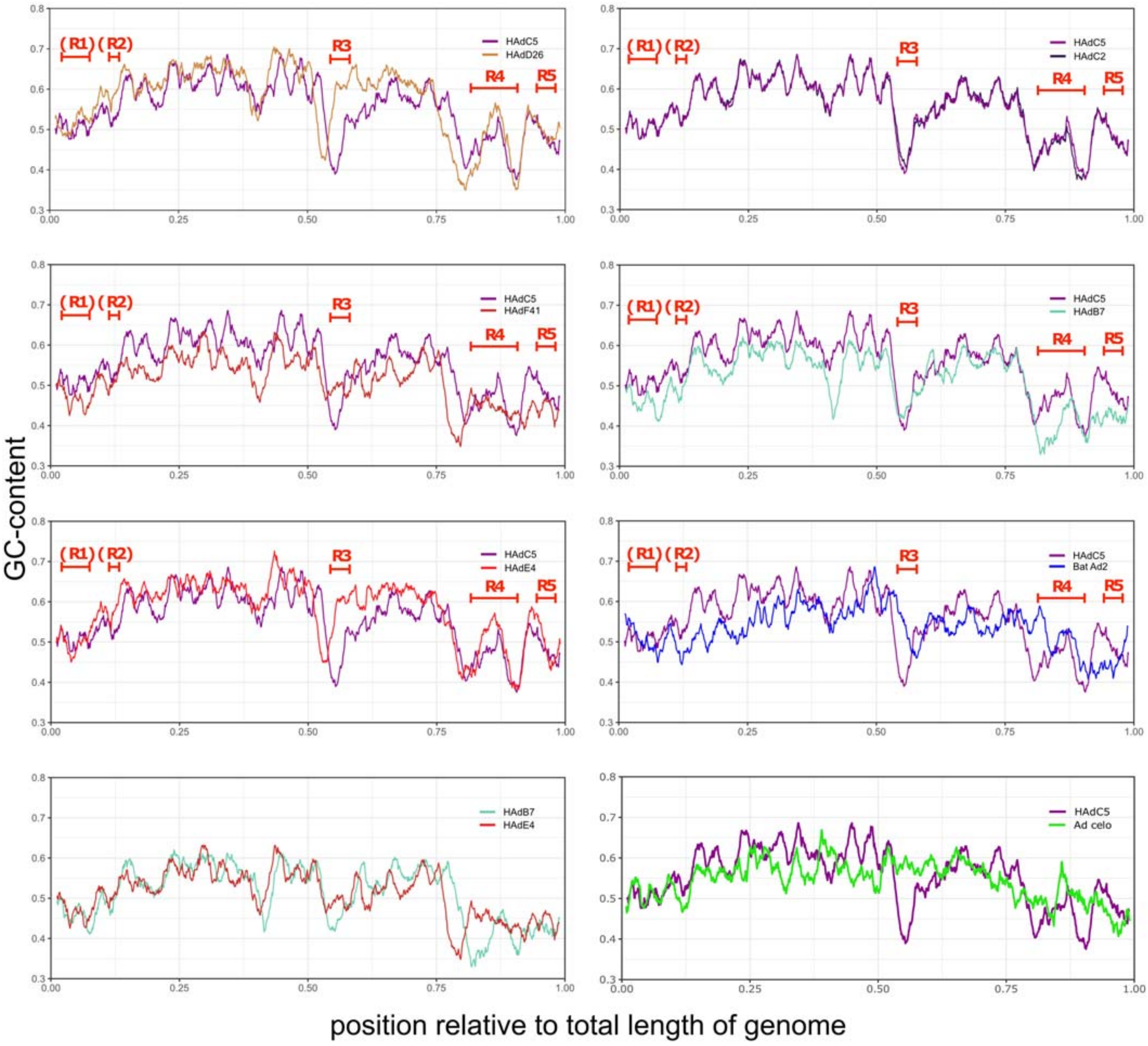

Figure EV5. Comparisons of GC-content distribution along various adenoviral genomes.

The Y-axis shows the GC-content. Because of the variable sizes of the tested genomes, the X-axis represents the relative positions along the entire length of the genome.
